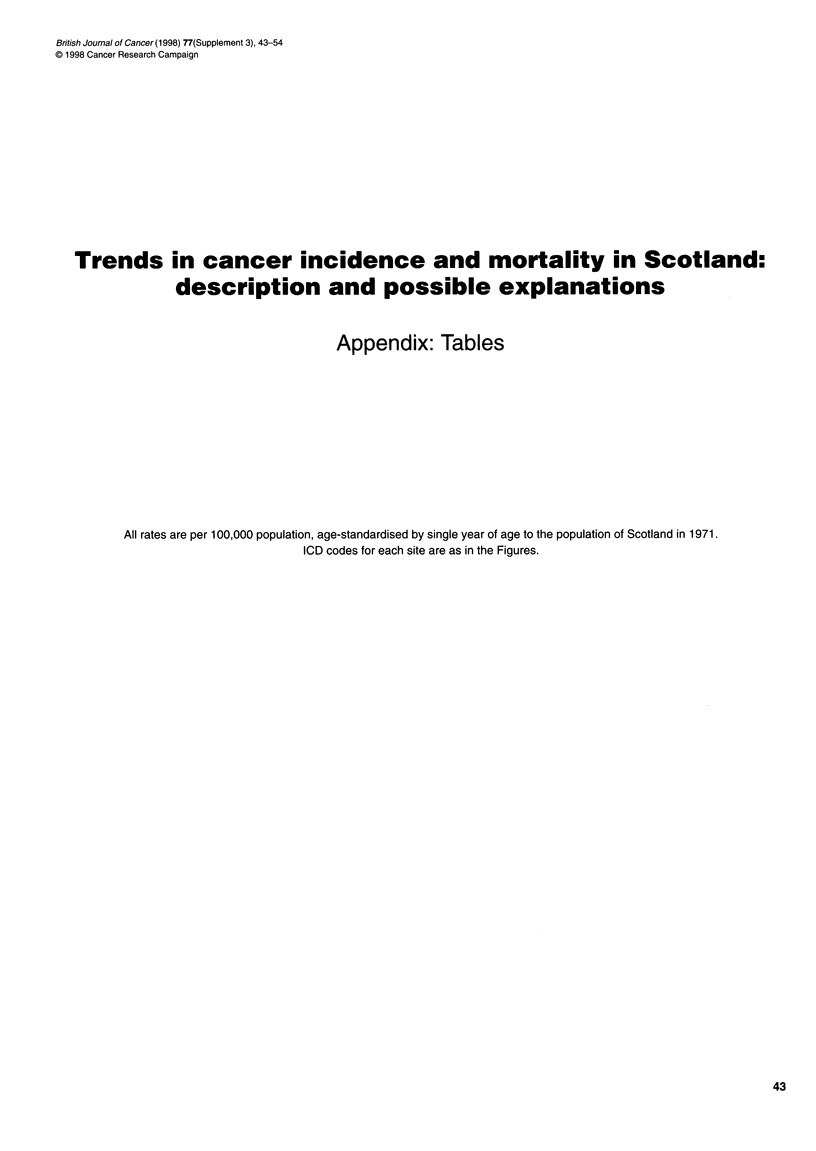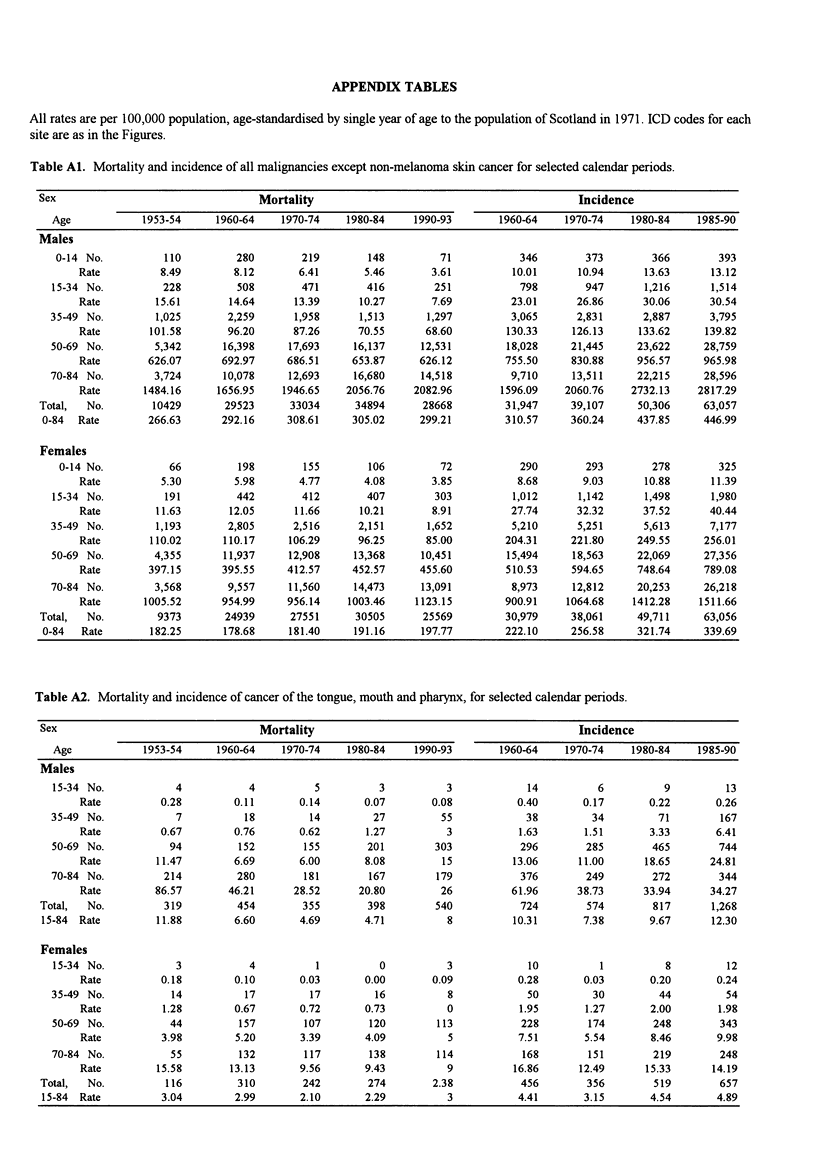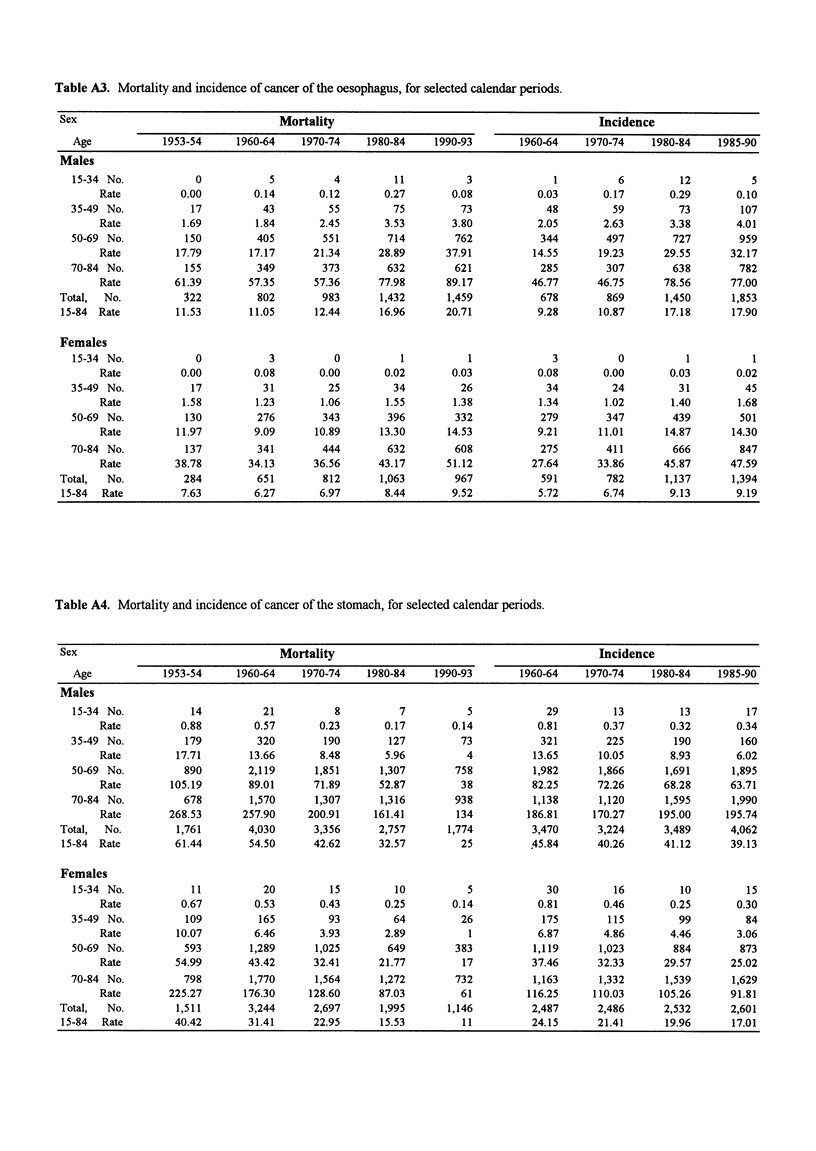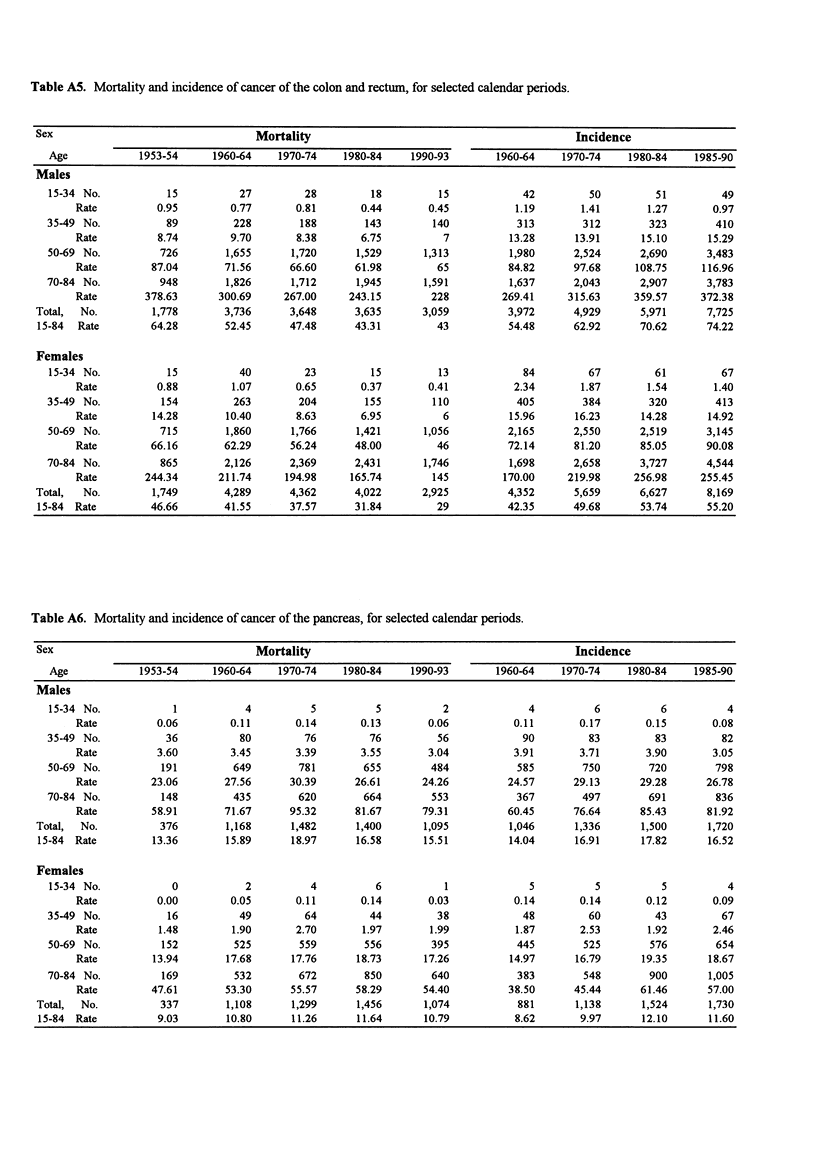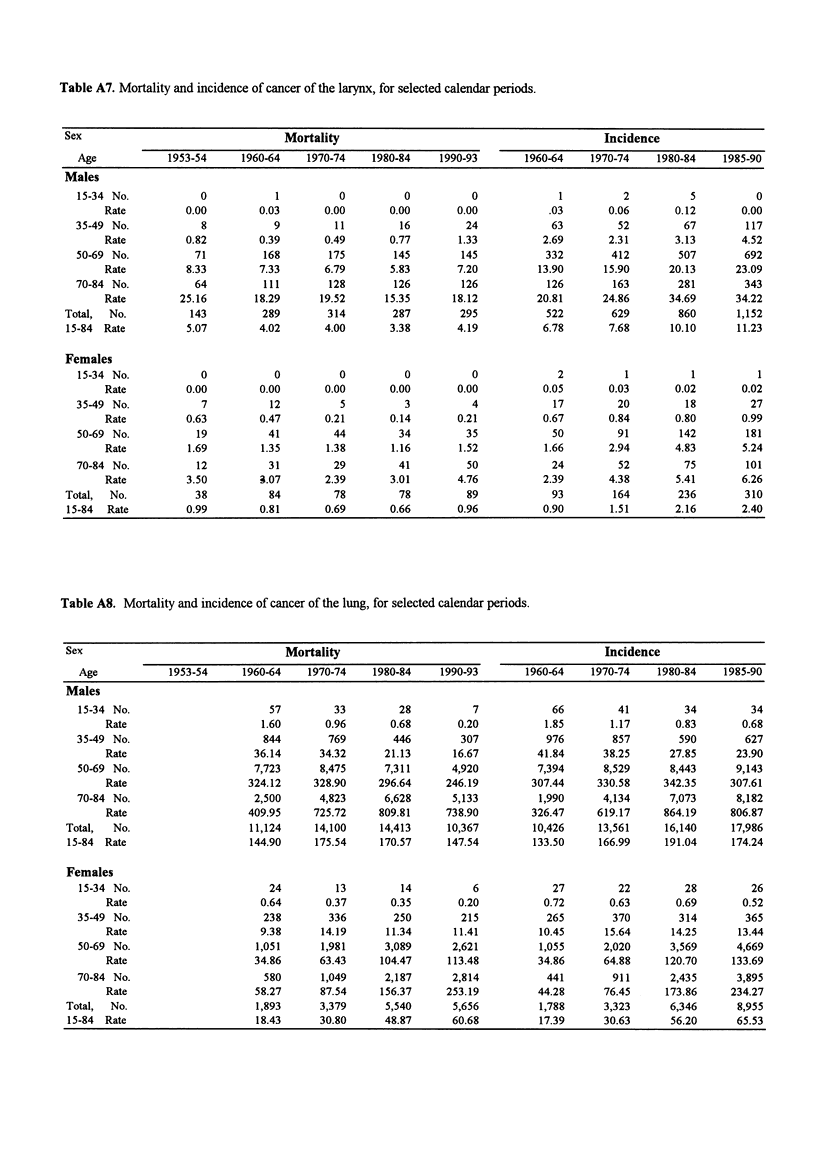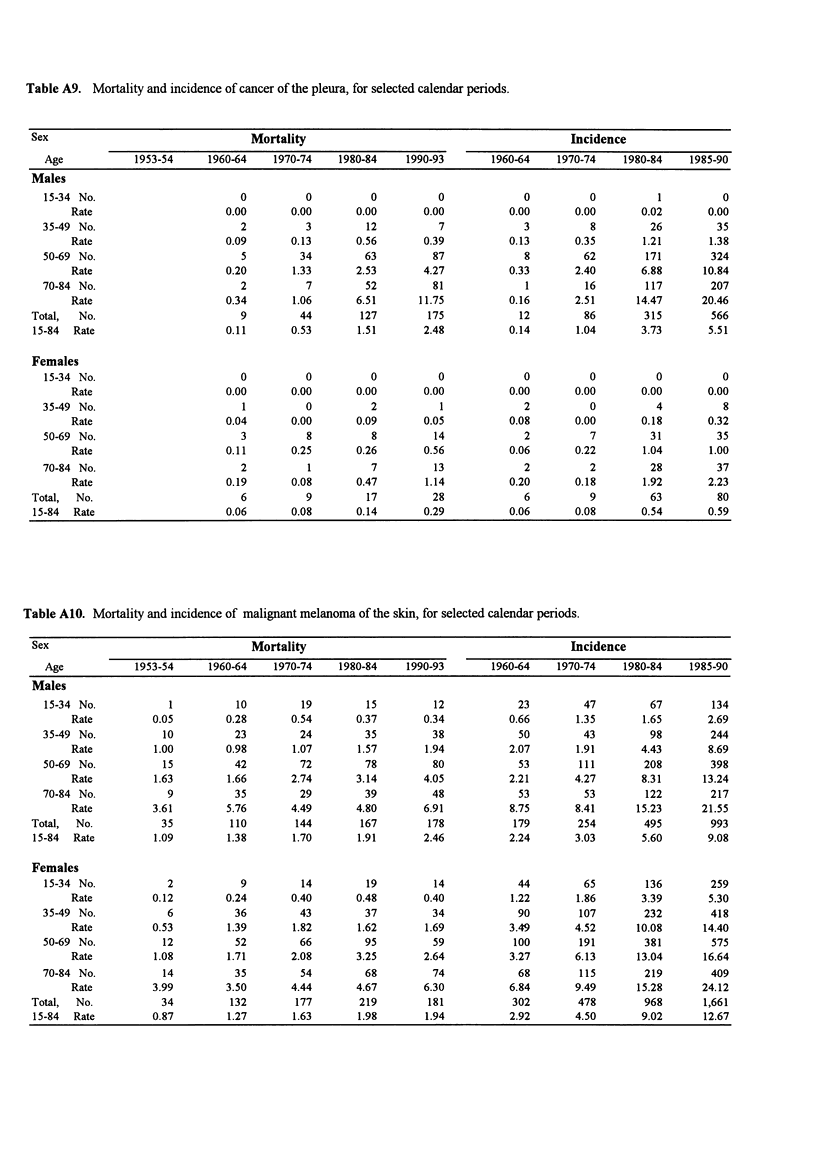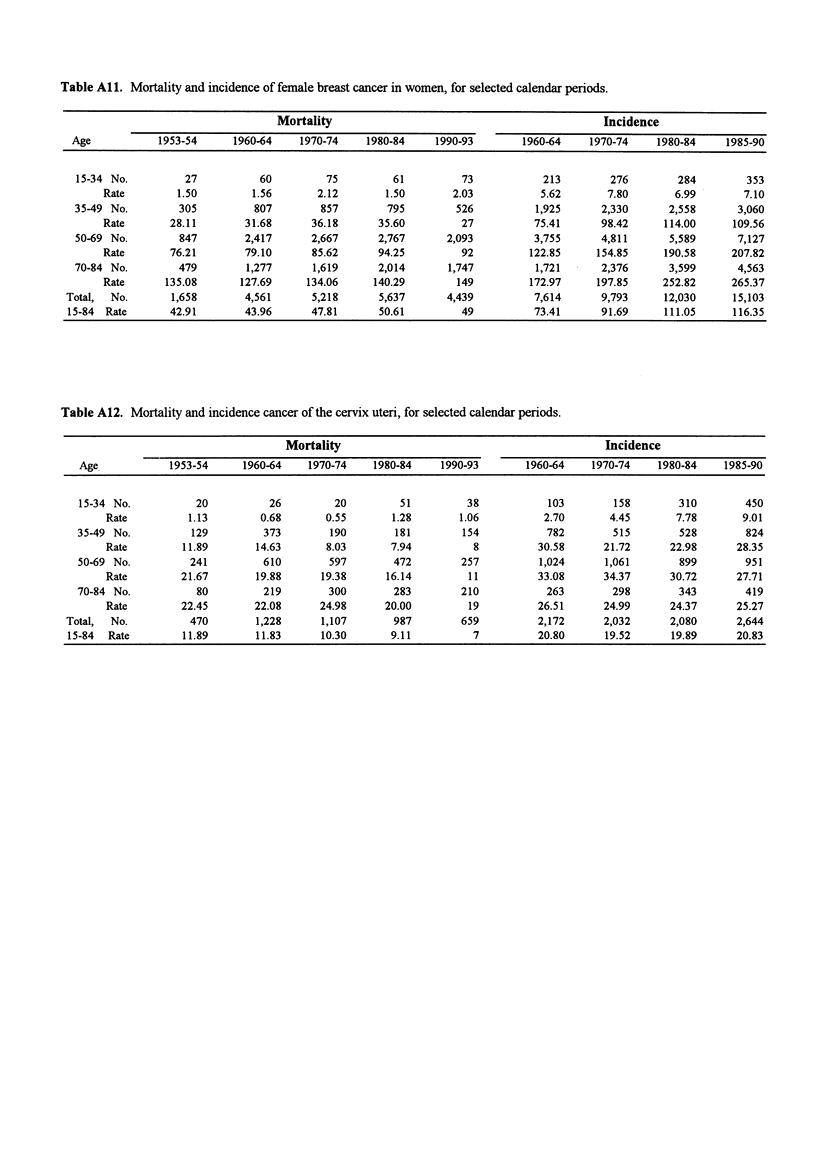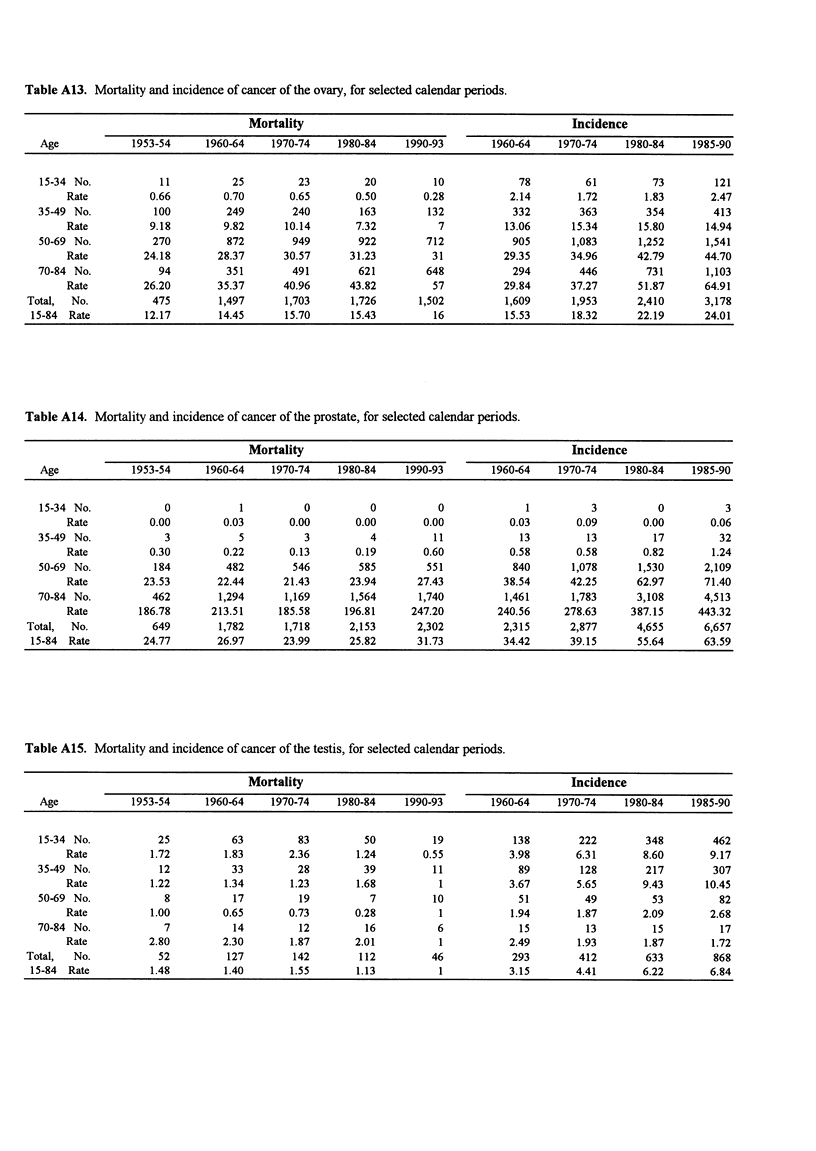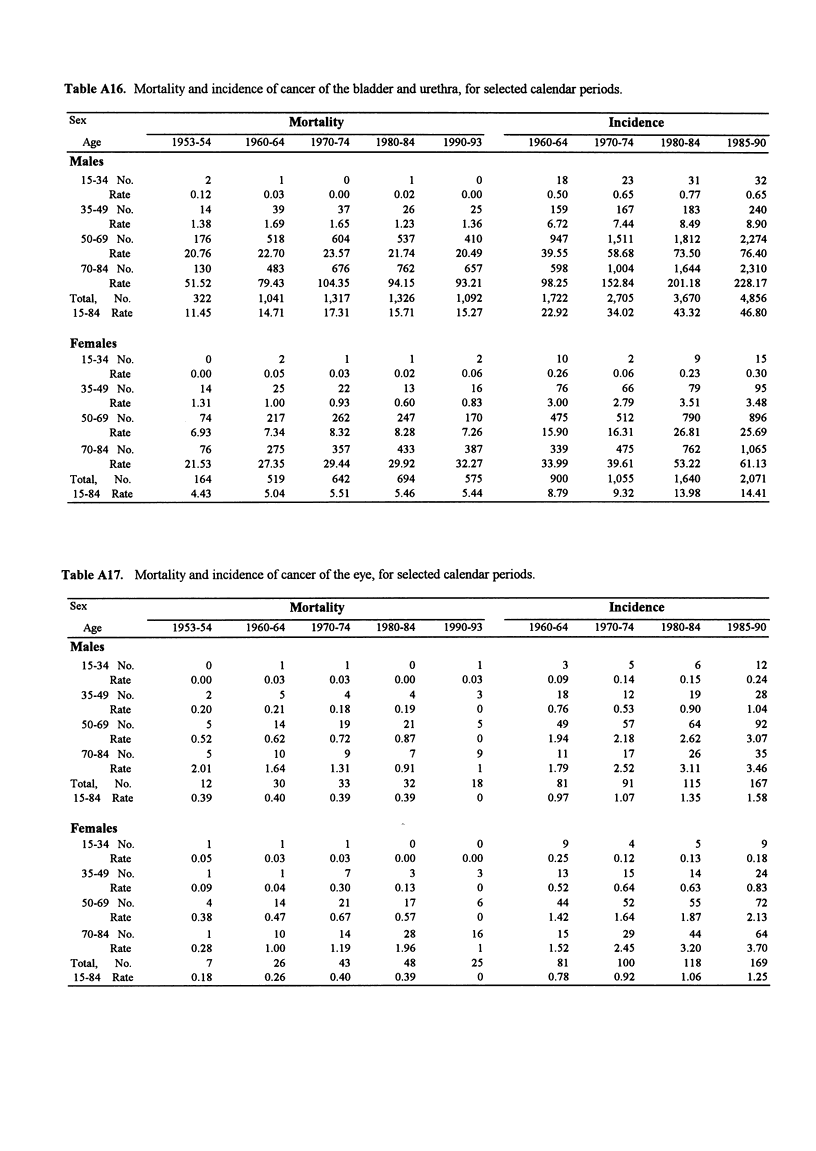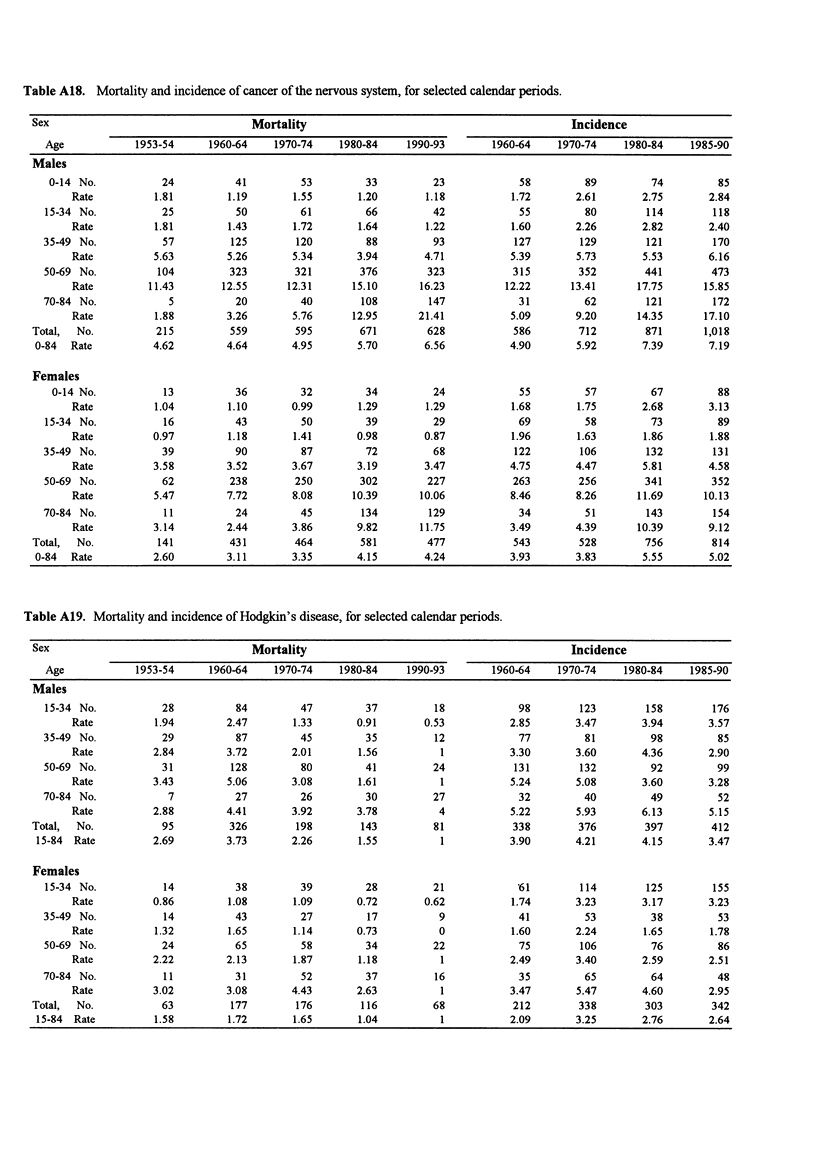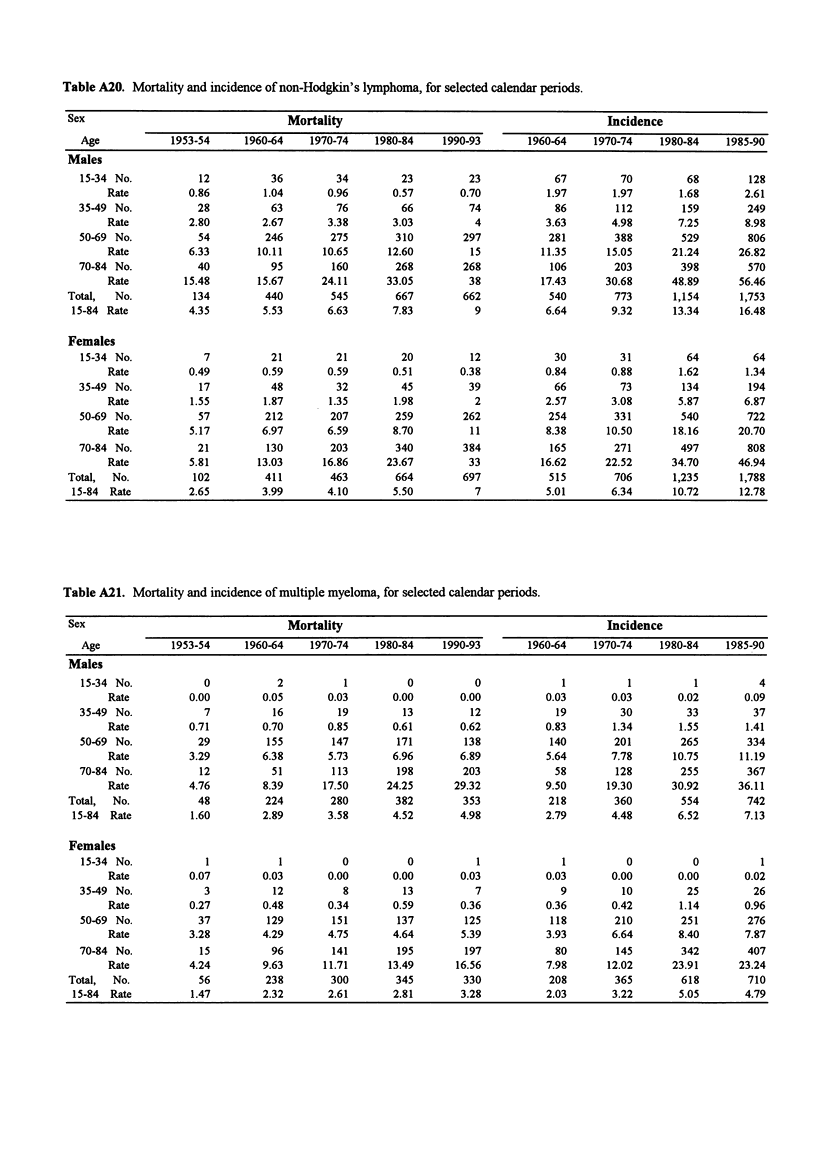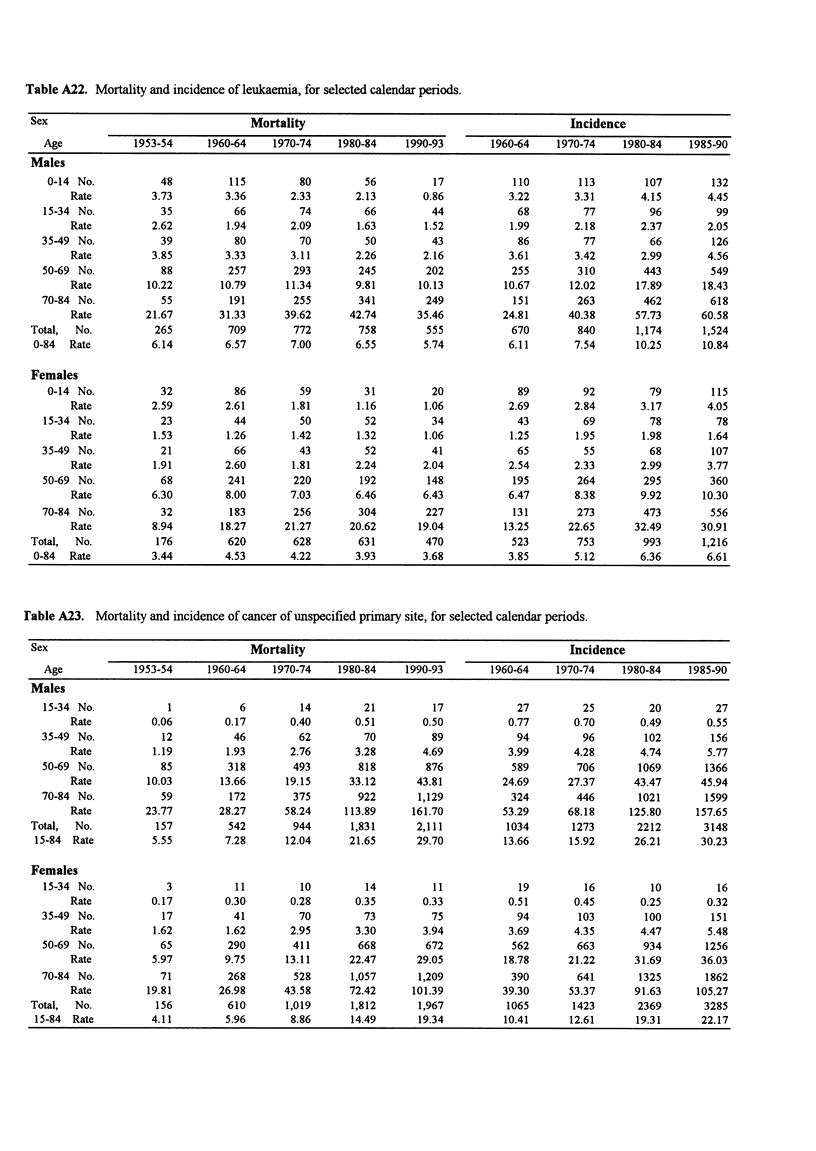# Trends in cancer incidence and mortality in Scotland: description and possible explanations

**Published:** 1998

**Authors:** 


British Journal of Cancer (1998) 77(Supplement 3), 43–54
© 1998 Cancer Research Campaign

### Trends in cancer incidence and mortality in Scotland: description and possible explanations

### Appendix: Tables

All rates are per 100,000 population, age-standardised by single year of age to the population of Scotland in 1971.
ICD codes for each site are as in the Figures.

### APPENDIX TABLES

All rates are per 100,000 population, age-standardised by single year of age to the population of Scotland in 1971. ICD codes for each site are as in the Figures.

**Table A1.** Mortality and incidence of all malignancies except non-melanoma skin cancer for selected calendar periods.

| Sex | Mortality | | | | | Incidence | | | |
|---|---|---|---|---|---|---|---|---|---|
| Age | 1953-54 | 1960-64 | 1970-74 | 1980-84 | 1990-93 | 1960-64 | 1970-74 | 1980-84 | 1985-90 |
| **Males** | | | | | | | | | |
| 0-14 No. | 110 | 280 | 219 | 148 | 71 | 346 | 373 | 366 | 393 |
| Rate | 8.49 | 8.12 | 6.41 | 5.46 | 3.61 | 10.01 | 10.94 | 13.63 | 13.12 |
| 15-34 No. | 228 | 508 | 471 | 416 | 251 | 798 | 947 | 1,216 | 1,514 |
| Rate | 15.61 | 14.64 | 13.39 | 10.27 | 7.69 | 23.01 | 26.86 | 30.06 | 30.54 |
| 35-49 No. | 1,025 | 2,259 | 1,958 | 1,513 | 1,297 | 3,065 | 2,831 | 2,887 | 3,795 |
| Rate | 101.58 | 96.20 | 87.26 | 70.55 | 68.60 | 130.33 | 126.13 | 133.62 | 139.82 |
| 50-69 No. | 5,342 | 16,398 | 17,693 | 16,137 | 12,531 | 18,028 | 21,445 | 23,622 | 28,759 |
| Rate | 626.07 | 692.97 | 686.51 | 653.87 | 626.12 | 755.50 | 830.88 | 956.57 | 965.98 |
| 70-84 No. | 3,724 | 10,078 | 12,693 | 16,680 | 14,518 | 9,710 | 13,511 | 22,215 | 28,596 |
| Rate | 1484.16 | 1656.95 | 1946.65 | 2056.76 | 2082.96 | 1596.09 | 2060.76 | 2732.13 | 2817.29 |
| Total, No. | 10429 | 29523 | 33034 | 34894 | 28668 | 31,947 | 39,107 | 50,306 | 63,057 |
| 0-84 Rate | 266.63 | 292.16 | 308.61 | 305.02 | 299.21 | 310.57 | 360.24 | 437.85 | 446.99 |
| **Females** | | | | | | | | | |
| 0-14 No. | 66 | 198 | 155 | 106 | 72 | 290 | 293 | 278 | 325 |
| Rate | 5.30 | 5.98 | 4.77 | 4.08 | 3.85 | 8.68 | 9.03 | 10.88 | 11.39 |
| 15-34 No. | 191 | 442 | 412 | 407 | 303 | 1,012 | 1,142 | 1,498 | 1,980 |
| Rate | 11.63 | 12.05 | 11.66 | 10.21 | 8.91 | 27.74 | 32.32 | 37.52 | 40.44 |
| 35-49 No. | 1,193 | 2,805 | 2,516 | 2,151 | 1,652 | 5,210 | 5,251 | 5,613 | 7,177 |
| Rate | 110.02 | 110.17 | 106.29 | 96.25 | 85.00 | 204.31 | 221.80 | 249.55 | 256.01 |
| 50-69 No. | 4,355 | 11,937 | 12,908 | 13,368 | 10,451 | 15,494 | 18,563 | 22,069 | 27,356 |
| Rate | 397.15 | 395.55 | 412.57 | 452.57 | 455.60 | 510.53 | 594.65 | 748.64 | 789.08 |
| 70-84 No. | 3,568 | 9,557 | 11,560 | 14,473 | 13,091 | 8,973 | 12,812 | 20,253 | 26,218 |
| Rate | 1005.52 | 954.99 | 956.14 | 1003.46 | 1123.15 | 900.91 | 1064.68 | 1412.28 | 1511.66 |
| Total, No. | 9373 | 24939 | 27551 | 30505 | 25569 | 30,979 | 38,061 | 49,711 | 63,056 |
| 0-84 Rate | 182.25 | 178.68 | 181.40 | 191.16 | 197.77 | 222.10 | 256.58 | 321.74 | 339.69 |

**Table A2.** Mortality and incidence of cancer of the tongue, mouth and pharynx, for selected calendar periods.

| Sex | Mortality | | | | | Incidence | | | |
|---|---|---|---|---|---|---|---|---|---|
| Age | 1953-54 | 1960-64 | 1970-74 | 1980-84 | 1990-93 | 1960-64 | 1970-74 | 1980-84 | 1985-90 |
| **Males** | | | | | | | | | |
| 15-34 No. | 4 | 4 | 5 | 3 | 3 | 14 | 6 | 9 | 13 |
| Rate | 0.28 | 0.11 | 0.14 | 0.07 | 0.08 | 0.40 | 0.17 | 0.22 | 0.26 |
| 35-49 No. | 7 | 18 | 14 | 27 | 55 | 38 | 34 | 71 | 167 |
| Rate | 0.67 | 0.76 | 0.62 | 1.27 | 3 | 1.63 | 1.51 | 3.33 | 6.41 |
| 50-69 No. | 94 | 152 | 155 | 201 | 303 | 296 | 285 | 465 | 744 |
| Rate | 11.47 | 6.69 | 6.00 | 8.08 | 15 | 13.06 | 11.00 | 18.65 | 24.81 |
| 70-84 No. | 214 | 280 | 181 | 167 | 179 | 376 | 249 | 272 | 344 |
| Rate | 86.57 | 46.21 | 28.52 | 20.80 | 26 | 61.96 | 38.73 | 33.94 | 34.27 |
| Total, No. | 319 | 454 | 355 | 398 | 540 | 724 | 574 | 817 | 1,268 |
| 15-84 Rate | 11.88 | 6.60 | 4.69 | 4.71 | 8 | 10.31 | 7.38 | 9.67 | 12.30 |
| **Females** | | | | | | | | | |
| 15-34 No. | 3 | 4 | 1 | 0 | 3 | 10 | 1 | 8 | 12 |
| Rate | 0.18 | 0.10 | 0.03 | 0.00 | 0.09 | 0.28 | 0.03 | 0.20 | 0.24 |
| 35-49 No. | 14 | 17 | 17 | 16 | 8 | 50 | 30 | 44 | 54 |
| Rate | 1.28 | 0.67 | 0.72 | 0.73 | 0 | 1.95 | 1.27 | 2.00 | 1.98 |
| 50-69 No. | 44 | 157 | 107 | 120 | 113 | 228 | 174 | 248 | 343 |
| Rate | 3.98 | 5.20 | 3.39 | 4.09 | 5 | 7.51 | 5.54 | 8.46 | 9.98 |
| 70-84 No. | 55 | 132 | 117 | 138 | 114 | 168 | 151 | 219 | 248 |
| Rate | 15.58 | 13.13 | 9.56 | 9.43 | 9 | 16.86 | 12.49 | 15.33 | 14.19 |
| Total, No. | 116 | 310 | 242 | 274 | 2.38 | 456 | 356 | 519 | 657 |
| 15-84 Rate | 3.04 | 2.99 | 2.10 | 2.29 | 3 | 4.41 | 3.15 | 4.54 | 4.89 |

**Table A3.** Mortality and incidence of cancer of the oesophagus, for selected calendar periods.

| Sex | Mortality | | | | | Incidence | | | |
|---|---|---|---|---|---|---|---|---|---|
| Age | 1953-54 | 1960-64 | 1970-74 | 1980-84 | 1990-93 | 1960-64 | 1970-74 | 1980-84 | 1985-90 |
| **Males** | | | | | | | | | |
| 15-34 No. | 0 | 5 | 4 | 11 | 3 | 1 | 6 | 12 | 5 |
| Rate | 0.00 | 0.14 | 0.12 | 0.27 | 0.08 | 0.03 | 0.17 | 0.29 | 0.10 |
| 35-49 No. | 17 | 43 | 55 | 75 | 73 | 48 | 59 | 73 | 107 |
| Rate | 1.69 | 1.84 | 2.45 | 3.53 | 3.80 | 2.05 | 2.63 | 3.38 | 4.01 |
| 50-69 No. | 150 | 405 | 551 | 714 | 762 | 344 | 497 | 727 | 959 |
| Rate | 17.79 | 17.17 | 21.34 | 28.89 | 37.91 | 14.55 | 19.23 | 29.55 | 32.17 |
| 70-84 No. | 155 | 349 | 373 | 632 | 621 | 285 | 307 | 638 | 782 |
| Rate | 61.39 | 57.35 | 57.36 | 77.98 | 89.17 | 46.77 | 46.75 | 78.56 | 77.00 |
| Total, No. | 322 | 802 | 983 | 1,432 | 1,459 | 678 | 869 | 1,450 | 1,853 |
| 15-84 Rate | 11.53 | 11.05 | 12.44 | 16.96 | 20.71 | 9.28 | 10.87 | 17.18 | 17.90 |
| **Females** | | | | | | | | | |
| 15-34 No. | 0 | 3 | 0 | 1 | 1 | 3 | 0 | 1 | 1 |
| Rate | 0.00 | 0.08 | 0.00 | 0.02 | 0.03 | 0.08 | 0.00 | 0.03 | 0.02 |
| 35-49 No. | 17 | 31 | 25 | 34 | 26 | 34 | 24 | 31 | 45 |
| Rate | 1.58 | 1.23 | 1.06 | 1.55 | 1.38 | 1.34 | 1.02 | 1.40 | 1.68 |
| 50-69 No. | 130 | 276 | 343 | 396 | 332 | 279 | 347 | 439 | 501 |
| Rate | 11.97 | 9.09 | 10.89 | 13.30 | 14.53 | 9.21 | 11.01 | 14.87 | 14.30 |
| 70-84 No. | 137 | 341 | 444 | 632 | 608 | 275 | 411 | 666 | 847 |
| Rate | 38.78 | 34.13 | 36.56 | 43.17 | 51.12 | 27.64 | 33.86 | 45.87 | 47.59 |
| Total, No. | 284 | 651 | 812 | 1,063 | 967 | 591 | 782 | 1,137 | 1,394 |
| 15-84 Rate | 7.63 | 6.27 | 6.97 | 8.44 | 9.52 | 5.72 | 6.74 | 9.13 | 9.19 |

**Table A4.** Mortality and incidence of cancer of the stomach, for selected calendar periods.

| Sex | Mortality | | | | | Incidence | | | |
|---|---|---|---|---|---|---|---|---|---|
| Age | 1953-54 | 1960-64 | 1970-74 | 1980-84 | 1990-93 | 1960-64 | 1970-74 | 1980-84 | 1985-90 |
| **Males** | | | | | | | | | |
| 15-34 No. | 14 | 21 | 8 | 7 | 5 | 29 | 13 | 13 | 17 |
| Rate | 0.88 | 0.57 | 0.23 | 0.17 | 0.14 | 0.81 | 0.37 | 0.32 | 0.34 |
| 35-49 No. | 179 | 320 | 190 | 127 | 73 | 321 | 225 | 190 | 160 |
| Rate | 17.71 | 13.66 | 8.48 | 5.96 | 4 | 13.65 | 10.05 | 8.93 | 6.02 |
| 50-69 No. | 890 | 2,119 | 1,851 | 1,307 | 758 | 1,982 | 1,866 | 1,691 | 1,895 |
| Rate | 105.19 | 89.01 | 71.89 | 52.87 | 38 | 82.25 | 72.26 | 68.28 | 63.71 |
| 70-84 No. | 678 | 1,570 | 1,307 | 1,316 | 938 | 1,138 | 1,120 | 1,595 | 1,990 |
| Rate | 268.53 | 257.90 | 200.91 | 161.41 | 134 | 186.81 | 170.27 | 195.00 | 195.74 |
| Total, No. | 1,761 | 4,030 | 3,356 | 2,757 | 1,774 | 3,470 | 3,224 | 3,489 | 4,062 |
| 15-84 Rate | 61.44 | 54.50 | 42.62 | 32.57 | 25 | 45.84 | 40.26 | 41.12 | 39.13 |
| **Females** | | | | | | | | | |
| 15-34 No. | 11 | 20 | 15 | 10 | 5 | 30 | 16 | 10 | 15 |
| Rate | 0.67 | 0.53 | 0.43 | 0.25 | 0.14 | 0.81 | 0.46 | 0.25 | 0.30 |
| 35-49 No. | 109 | 165 | 93 | 64 | 26 | 175 | 115 | 99 | 84 |
| Rate | 10.07 | 6.46 | 3.93 | 2.89 | 1 | 6.87 | 4.86 | 4.46 | 3.06 |
| 50-69 No. | 593 | 1,289 | 1,025 | 649 | 383 | 1,119 | 1,023 | 884 | 873 |
| Rate | 54.99 | 43.42 | 32.41 | 21.77 | 17 | 37.46 | 32.33 | 29.57 | 25.02 |
| 70-84 No. | 798 | 1,770 | 1,564 | 1,272 | 732 | 1,163 | 1,332 | 1,539 | 1,629 |
| Rate | 225.27 | 176.30 | 128.60 | 87.03 | 61 | 116.25 | 110.03 | 105.26 | 91.81 |
| Total, No. | 1,511 | 3,244 | 2,697 | 1,995 | 1,146 | 2,487 | 2,486 | 2,532 | 2,601 |
| 15-84 Rate | 40.42 | 31.41 | 22.95 | 15.53 | 11 | 24.15 | 21.41 | 19.96 | 17.01 |

**Table A5.** Mortality and incidence of cancer of the colon and rectum, for selected calendar periods.

| Sex | | Mortality | | | | | Incidence | | | |
|---|---|---|---|---|---|---|---|---|---|---|
| Age | | 1953-54 | 1960-64 | 1970-74 | 1980-84 | 1990-93 | 1960-64 | 1970-74 | 1980-84 | 1985-90 |
| **Males** | | | | | | | | | | |
| 15-34 | No. | 15 | 27 | 28 | 18 | 15 | 42 | 50 | 51 | 49 |
| | Rate | 0.95 | 0.77 | 0.81 | 0.44 | 0.45 | 1.19 | 1.41 | 1.27 | 0.97 |
| 35-49 | No. | 89 | 228 | 188 | 143 | 140 | 313 | 312 | 323 | 410 |
| | Rate | 8.74 | 9.70 | 8.38 | 6.75 | 7 | 13.28 | 13.91 | 15.10 | 15.29 |
| 50-69 | No. | 726 | 1,655 | 1,720 | 1,529 | 1,313 | 1,980 | 2,524 | 2,690 | 3,483 |
| | Rate | 87.04 | 71.56 | 66.60 | 61.98 | 65 | 84.82 | 97.68 | 108.75 | 116.96 |
| 70-84 | No. | 948 | 1,826 | 1,712 | 1,945 | 1,591 | 1,637 | 2,043 | 2,907 | 3,783 |
| | Rate | 378.63 | 300.69 | 267.00 | 243.15 | 228 | 269.41 | 315.63 | 359.57 | 372.38 |
| Total, | No. | 1,778 | 3,736 | 3,648 | 3,635 | 3,059 | 3,972 | 4,929 | 5,971 | 7,725 |
| 15-84 | Rate | 64.28 | 52.45 | 47.48 | 43.31 | 43 | 54.48 | 62.92 | 70.62 | 74.22 |
| **Females** | | | | | | | | | | |
| 15-34 | No. | 15 | 40 | 23 | 15 | 13 | 84 | 67 | 61 | 67 |
| | Rate | 0.88 | 1.07 | 0.65 | 0.37 | 0.41 | 2.34 | 1.87 | 1.54 | 1.40 |
| 35-49 | No. | 154 | 263 | 204 | 155 | 110 | 405 | 384 | 320 | 413 |
| | Rate | 14.28 | 10.40 | 8.63 | 6.95 | 6 | 15.96 | 16.23 | 14.28 | 14.92 |
| 50-69 | No. | 715 | 1,860 | 1,766 | 1,421 | 1,056 | 2,165 | 2,550 | 2,519 | 3,145 |
| | Rate | 66.16 | 62.29 | 56.24 | 48.00 | 46 | 72.14 | 81.20 | 85.05 | 90.08 |
| 70-84 | No. | 865 | 2,126 | 2,369 | 2,431 | 1,746 | 1,698 | 2,658 | 3,727 | 4,544 |
| | Rate | 244.34 | 211.74 | 194.98 | 165.74 | 145 | 170.00 | 219.98 | 256.98 | 255.45 |
| Total, | No. | 1,749 | 4,289 | 4,362 | 4,022 | 2,925 | 4,352 | 5,659 | 6,627 | 8,169 |
| 15-84 | Rate | 46.66 | 41.55 | 37.57 | 31.84 | 29 | 42.35 | 49.68 | 53.74 | 55.20 |

**Table A6.** Mortality and incidence of cancer of the pancreas, for selected calendar periods.

| Sex | | Mortality | | | | | Incidence | | | |
|---|---|---|---|---|---|---|---|---|---|---|
| Age | | 1953-54 | 1960-64 | 1970-74 | 1980-84 | 1990-93 | 1960-64 | 1970-74 | 1980-84 | 1985-90 |
| **Males** | | | | | | | | | | |
| 15-34 | No. | 1 | 4 | 5 | 5 | 2 | 4 | 6 | 6 | 4 |
| | Rate | 0.06 | 0.11 | 0.14 | 0.13 | 0.06 | 0.11 | 0.17 | 0.15 | 0.08 |
| 35-49 | No. | 36 | 80 | 76 | 76 | 56 | 90 | 83 | 83 | 82 |
| | Rate | 3.60 | 3.45 | 3.39 | 3.55 | 3.04 | 3.91 | 3.71 | 3.90 | 3.05 |
| 50-69 | No. | 191 | 649 | 781 | 655 | 484 | 585 | 750 | 720 | 798 |
| | Rate | 23.06 | 27.56 | 30.39 | 26.61 | 24.26 | 24.57 | 29.13 | 29.28 | 26.78 |
| 70-84 | No. | 148 | 435 | 620 | 664 | 553 | 367 | 497 | 691 | 836 |
| | Rate | 58.91 | 71.67 | 95.32 | 81.67 | 79.31 | 60.45 | 76.64 | 85.43 | 81.92 |
| Total, | No. | 376 | 1,168 | 1,482 | 1,400 | 1,095 | 1,046 | 1,336 | 1,500 | 1,720 |
| 15-84 | Rate | 13.36 | 15.89 | 18.97 | 16.58 | 15.51 | 14.04 | 16.91 | 17.82 | 16.52 |
| **Females** | | | | | | | | | | |
| 15-34 | No. | 0 | 2 | 4 | 6 | 1 | 5 | 5 | 5 | 4 |
| | Rate | 0.00 | 0.05 | 0.11 | 0.14 | 0.03 | 0.14 | 0.14 | 0.12 | 0.09 |
| 35-49 | No. | 16 | 49 | 64 | 44 | 38 | 48 | 60 | 43 | 67 |
| | Rate | 1.48 | 1.90 | 2.70 | 1.97 | 1.99 | 1.87 | 2.53 | 1.92 | 2.46 |
| 50-69 | No. | 152 | 525 | 559 | 556 | 395 | 445 | 525 | 576 | 654 |
| | Rate | 13.94 | 17.68 | 17.76 | 18.73 | 17.26 | 14.97 | 16.79 | 19.35 | 18.67 |
| 70-84 | No. | 169 | 532 | 672 | 850 | 640 | 383 | 548 | 900 | 1,005 |
| | Rate | 47.61 | 53.30 | 55.57 | 58.29 | 54.40 | 38.50 | 45.44 | 61.46 | 57.00 |
| Total, | No. | 337 | 1,108 | 1,299 | 1,456 | 1,074 | 881 | 1,138 | 1,524 | 1,730 |
| 15-84 | Rate | 9.03 | 10.80 | 11.26 | 11.64 | 10.79 | 8.62 | 9.97 | 12.10 | 11.60 |

**Table A7.** Mortality and incidence of cancer of the larynx, for selected calendar periods.

| Sex | | Mortality | | | | | Incidence | | | |
|---|---|---|---|---|---|---|---|---|---|---|
| Age | | 1953-54 | 1960-64 | 1970-74 | 1980-84 | 1990-93 | 1960-64 | 1970-74 | 1980-84 | 1985-90 |
| **Males** | | | | | | | | | | |
| 15-34 | No. | 0 | 1 | 0 | 0 | 0 | 1 | 2 | 5 | 0 |
| | Rate | 0.00 | 0.03 | 0.00 | 0.00 | 0.00 | .03 | 0.06 | 0.12 | 0.00 |
| 35-49 | No. | 8 | 9 | 11 | 16 | 24 | 63 | 52 | 67 | 117 |
| | Rate | 0.82 | 0.39 | 0.49 | 0.77 | 1.33 | 2.69 | 2.31 | 3.13 | 4.52 |
| 50-69 | No. | 71 | 168 | 175 | 145 | 145 | 332 | 412 | 507 | 692 |
| | Rate | 8.33 | 7.33 | 6.79 | 5.83 | 7.20 | 13.90 | 15.90 | 20.13 | 23.09 |
| 70-84 | No. | 64 | 111 | 128 | 126 | 126 | 126 | 163 | 281 | 343 |
| | Rate | 25.16 | 18.29 | 19.52 | 15.35 | 18.12 | 20.81 | 24.86 | 34.69 | 34.22 |
| Total, | No. | 143 | 289 | 314 | 287 | 295 | 522 | 629 | 860 | 1,152 |
| 15-84 | Rate | 5.07 | 4.02 | 4.00 | 3.38 | 4.19 | 6.78 | 7.68 | 10.10 | 11.23 |
| **Females** | | | | | | | | | | |
| 15-34 | No. | 0 | 0 | 0 | 0 | 0 | 2 | 1 | 1 | 1 |
| | Rate | 0.00 | 0.00 | 0.00 | 0.00 | 0.00 | 0.05 | 0.03 | 0.02 | 0.02 |
| 35-49 | No. | 7 | 12 | 5 | 3 | 4 | 17 | 20 | 18 | 27 |
| | Rate | 0.63 | 0.47 | 0.21 | 0.14 | 0.21 | 0.67 | 0.84 | 0.80 | 0.99 |
| 50-69 | No. | 19 | 41 | 44 | 34 | 35 | 50 | 91 | 142 | 181 |
| | Rate | 1.69 | 1.35 | 1.38 | 1.16 | 1.52 | 1.66 | 2.94 | 4.83 | 5.24 |
| 70-84 | No. | 12 | 31 | 29 | 41 | 50 | 24 | 52 | 75 | 101 |
| | Rate | 3.50 | 3.07 | 2.39 | 3.01 | 4.76 | 2.39 | 4.38 | 5.41 | 6.26 |
| Total, | No. | 38 | 84 | 78 | 78 | 89 | 93 | 164 | 236 | 310 |
| 15-84 | Rate | 0.99 | 0.81 | 0.69 | 0.66 | 0.96 | 0.90 | 1.51 | 2.16 | 2.40 |

**Table A8.** Mortality and incidence of cancer of the lung, for selected calendar periods.

| Sex | | Mortality | | | | | Incidence | | | |
|---|---|---|---|---|---|---|---|---|---|---|
| Age | | 1953-54 | 1960-64 | 1970-74 | 1980-84 | 1990-93 | 1960-64 | 1970-74 | 1980-84 | 1985-90 |
| **Males** | | | | | | | | | | |
| 15-34 | No. | | 57 | 33 | 28 | 7 | 66 | 41 | 34 | 34 |
| | Rate | | 1.60 | 0.96 | 0.68 | 0.20 | 1.85 | 1.17 | 0.83 | 0.68 |
| 35-49 | No. | | 844 | 769 | 446 | 307 | 976 | 857 | 590 | 627 |
| | Rate | | 36.14 | 34.32 | 21.13 | 16.67 | 41.84 | 38.25 | 27.85 | 23.90 |
| 50-69 | No. | | 7,723 | 8,475 | 7,311 | 4,920 | 7,394 | 8,529 | 8,443 | 9,143 |
| | Rate | | 324.12 | 328.90 | 296.64 | 246.19 | 307.44 | 330.58 | 342.35 | 307.61 |
| 70-84 | No. | | 2,500 | 4,823 | 6,628 | 5,133 | 1,990 | 4,134 | 7,073 | 8,182 |
| | Rate | | 409.95 | 725.72 | 809.81 | 738.90 | 326.47 | 619.17 | 864.19 | 806.87 |
| Total, | No. | | 11,124 | 14,100 | 14,413 | 10,367 | 10,426 | 13,561 | 16,140 | 17,986 |
| 15-84 | Rate | | 144.90 | 175.54 | 170.57 | 147.54 | 133.50 | 166.99 | 191.04 | 174.24 |
| **Females** | | | | | | | | | | |
| 15-34 | No. | | 24 | 13 | 14 | 6 | 27 | 22 | 28 | 26 |
| | Rate | | 0.64 | 0.37 | 0.35 | 0.20 | 0.72 | 0.63 | 0.69 | 0.52 |
| 35-49 | No. | | 238 | 336 | 250 | 215 | 265 | 370 | 314 | 365 |
| | Rate | | 9.38 | 14.19 | 11.34 | 11.41 | 10.45 | 15.64 | 14.25 | 13.44 |
| 50-69 | No. | | 1,051 | 1,981 | 3,089 | 2,621 | 1,055 | 2,020 | 3,569 | 4,669 |
| | Rate | | 34.86 | 63.43 | 104.47 | 113.48 | 34.86 | 64.88 | 120.70 | 133.69 |
| 70-84 | No. | | 580 | 1,049 | 2,187 | 2,814 | 441 | 911 | 2,435 | 3,895 |
| | Rate | | 58.27 | 87.54 | 156.37 | 253.19 | 44.28 | 76.45 | 173.86 | 234.27 |
| Total, | No. | | 1,893 | 3,379 | 5,540 | 5,656 | 1,788 | 3,323 | 6,346 | 8,955 |
| 15-84 | Rate | | 18.43 | 30.80 | 48.87 | 60.68 | 17.39 | 30.63 | 56.20 | 65.53 |

**Table A9.** Mortality and incidence of cancer of the pleura, for selected calendar periods.

| Sex | | Mortality | | | | | Incidence | | | |
|---|---|---|---|---|---|---|---|---|---|---|
| Age | | 1953-54 | 1960-64 | 1970-74 | 1980-84 | 1990-93 | 1960-64 | 1970-74 | 1980-84 | 1985-90 |
| **Males** | | | | | | | | | | |
| 15-34 | No. | | 0 | 0 | 0 | 0 | 0 | 0 | 1 | 0 |
| | Rate | | 0.00 | 0.00 | 0.00 | 0.00 | 0.00 | 0.00 | 0.02 | 0.00 |
| 35-49 | No. | | 2 | 3 | 12 | 7 | 3 | 8 | 26 | 35 |
| | Rate | | 0.09 | 0.13 | 0.56 | 0.39 | 0.13 | 0.35 | 1.21 | 1.38 |
| 50-69 | No. | | 5 | 34 | 63 | 87 | 8 | 62 | 171 | 324 |
| | Rate | | 0.20 | 1.33 | 2.53 | 4.27 | 0.33 | 2.40 | 6.88 | 10.84 |
| 70-84 | No. | | 2 | 7 | 52 | 81 | 1 | 16 | 117 | 207 |
| | Rate | | 0.34 | 1.06 | 6.51 | 11.75 | 0.16 | 2.51 | 14.47 | 20.46 |
| Total, | No. | | 9 | 44 | 127 | 175 | 12 | 86 | 315 | 566 |
| 15-84 | Rate | | 0.11 | 0.53 | 1.51 | 2.48 | 0.14 | 1.04 | 3.73 | 5.51 |
| **Females** | | | | | | | | | | |
| 15-34 | No. | | 0 | 0 | 0 | 0 | 0 | 0 | 0 | 0 |
| | Rate | | 0.00 | 0.00 | 0.00 | 0.00 | 0.00 | 0.00 | 0.00 | 0.00 |
| 35-49 | No. | | 1 | 0 | 2 | 1 | 2 | 0 | 4 | 8 |
| | Rate | | 0.04 | 0.00 | 0.09 | 0.05 | 0.08 | 0.00 | 0.18 | 0.32 |
| 50-69 | No. | | 3 | 8 | 8 | 14 | 2 | 7 | 31 | 35 |
| | Rate | | 0.11 | 0.25 | 0.26 | 0.56 | 0.06 | 0.22 | 1.04 | 1.00 |
| 70-84 | No. | | 2 | 1 | 7 | 13 | 2 | 2 | 28 | 37 |
| | Rate | | 0.19 | 0.08 | 0.47 | 1.14 | 0.20 | 0.18 | 1.92 | 2.23 |
| Total, | No. | | 6 | 9 | 17 | 28 | 6 | 9 | 63 | 80 |
| 15-84 | Rate | | 0.06 | 0.08 | 0.14 | 0.29 | 0.06 | 0.08 | 0.54 | 0.59 |

**Table A10.** Mortality and incidence of malignant melanoma of the skin, for selected calendar periods.

| Sex | | Mortality | | | | | Incidence | | | |
|---|---|---|---|---|---|---|---|---|---|---|
| Age | | 1953-54 | 1960-64 | 1970-74 | 1980-84 | 1990-93 | 1960-64 | 1970-74 | 1980-84 | 1985-90 |
| **Males** | | | | | | | | | | |
| 15-34 | No. | 1 | 10 | 19 | 15 | 12 | 23 | 47 | 67 | 134 |
| | Rate | 0.05 | 0.28 | 0.54 | 0.37 | 0.34 | 0.66 | 1.35 | 1.65 | 2.69 |
| 35-49 | No. | 10 | 23 | 24 | 35 | 38 | 50 | 43 | 98 | 244 |
| | Rate | 1.00 | 0.98 | 1.07 | 1.57 | 1.94 | 2.07 | 1.91 | 4.43 | 8.69 |
| 50-69 | No. | 15 | 42 | 72 | 78 | 80 | 53 | 111 | 208 | 398 |
| | Rate | 1.63 | 1.66 | 2.74 | 3.14 | 4.05 | 2.21 | 4.27 | 8.31 | 13.24 |
| 70-84 | No. | 9 | 35 | 29 | 39 | 48 | 53 | 53 | 122 | 217 |
| | Rate | 3.61 | 5.76 | 4.49 | 4.80 | 6.91 | 8.75 | 8.41 | 15.23 | 21.55 |
| Total, | No. | 35 | 110 | 144 | 167 | 178 | 179 | 254 | 495 | 993 |
| 15-84 | Rate | 1.09 | 1.38 | 1.70 | 1.91 | 2.46 | 2.24 | 3.03 | 5.60 | 9.08 |
| **Females** | | | | | | | | | | |
| 15-34 | No. | 2 | 9 | 14 | 19 | 14 | 44 | 65 | 136 | 259 |
| | Rate | 0.12 | 0.24 | 0.40 | 0.48 | 0.40 | 1.22 | 1.86 | 3.39 | 5.30 |
| 35-49 | No. | 6 | 36 | 43 | 37 | 34 | 90 | 107 | 232 | 418 |
| | Rate | 0.53 | 1.39 | 1.82 | 1.62 | 1.69 | 3.49 | 4.52 | 10.08 | 14.40 |
| 50-69 | No. | 12 | 52 | 66 | 95 | 59 | 100 | 191 | 381 | 575 |
| | Rate | 1.08 | 1.71 | 2.08 | 3.25 | 2.64 | 3.27 | 6.13 | 13.04 | 16.64 |
| 70-84 | No. | 14 | 35 | 54 | 68 | 74 | 68 | 115 | 219 | 409 |
| | Rate | 3.99 | 3.50 | 4.44 | 4.67 | 6.30 | 6.84 | 9.49 | 15.28 | 24.12 |
| Total, | No. | 34 | 132 | 177 | 219 | 181 | 302 | 478 | 968 | 1,661 |
| 15-84 | Rate | 0.87 | 1.27 | 1.63 | 1.98 | 1.94 | 2.92 | 4.50 | 9.02 | 12.67 |

**Table A11.** Mortality and incidence of female breast cancer in women, for selected calendar periods.

| Age | | Mortality | | | | | Incidence | | | |
|---|---|---|---|---|---|---|---|---|---|---|
| | | 1953-54 | 1960-64 | 1970-74 | 1980-84 | 1990-93 | 1960-64 | 1970-74 | 1980-84 | 1985-90 |
| 15-34 | No. | 27 | 60 | 75 | 61 | 73 | 213 | 276 | 284 | 353 |
| | Rate | 1.50 | 1.56 | 2.12 | 1.50 | 2.03 | 5.62 | 7.80 | 6.99 | 7.10 |
| 35-49 | No. | 305 | 807 | 857 | 795 | 526 | 1,925 | 2,330 | 2,558 | 3,060 |
| | Rate | 28.11 | 31.68 | 36.18 | 35.60 | 27 | 75.41 | 98.42 | 114.00 | 109.56 |
| 50-69 | No. | 847 | 2,417 | 2,667 | 2,767 | 2,093 | 3,755 | 4,811 | 5,589 | 7,127 |
| | Rate | 76.21 | 79.10 | 85.62 | 94.25 | 92 | 122.85 | 154.85 | 190.58 | 207.82 |
| 70-84 | No. | 479 | 1,277 | 1,619 | 2,014 | 1,747 | 1,721 | 2,376 | 3,599 | 4,563 |
| | Rate | 135.08 | 127.69 | 134.06 | 140.29 | 149 | 172.97 | 197.85 | 252.82 | 265.37 |
| Total, | No. | 1,658 | 4,561 | 5,218 | 5,637 | 4,439 | 7,614 | 9,793 | 12,030 | 15,103 |
| 15-84 | Rate | 42.91 | 43.96 | 47.81 | 50.61 | 49 | 73.41 | 91.69 | 111.05 | 116.35 |

**Table A12.** Mortality and incidence cancer of the cervix uteri, for selected calendar periods.

| Age | | Mortality | | | | | Incidence | | | |
|---|---|---|---|---|---|---|---|---|---|---|
| | | 1953-54 | 1960-64 | 1970-74 | 1980-84 | 1990-93 | 1960-64 | 1970-74 | 1980-84 | 1985-90 |
| 15-34 | No. | 20 | 26 | 20 | 51 | 38 | 103 | 158 | 310 | 450 |
| | Rate | 1.13 | 0.68 | 0.55 | 1.28 | 1.06 | 2.70 | 4.45 | 7.78 | 9.01 |
| 35-49 | No. | 129 | 373 | 190 | 181 | 154 | 782 | 515 | 528 | 824 |
| | Rate | 11.89 | 14.63 | 8.03 | 7.94 | 8 | 30.58 | 21.72 | 22.98 | 28.35 |
| 50-69 | No. | 241 | 610 | 597 | 472 | 257 | 1,024 | 1,061 | 899 | 951 |
| | Rate | 21.67 | 19.88 | 19.38 | 16.14 | 11 | 33.08 | 34.37 | 30.72 | 27.71 |
| 70-84 | No. | 80 | 219 | 300 | 283 | 210 | 263 | 298 | 343 | 419 |
| | Rate | 22.45 | 22.08 | 24.98 | 20.00 | 19 | 26.51 | 24.99 | 24.37 | 25.27 |
| Total, | No. | 470 | 1,228 | 1,107 | 987 | 659 | 2,172 | 2,032 | 2,080 | 2,644 |
| 15-84 | Rate | 11.89 | 11.83 | 10.30 | 9.11 | 7 | 20.80 | 19.52 | 19.89 | 20.83 |

**Table A13.** Mortality and incidence of cancer of the ovary, for selected calendar periods.

| Age | Mortality | | | | | Incidence | | | |
|---|---|---|---|---|---|---|---|---|---|
| | 1953-54 | 1960-64 | 1970-74 | 1980-84 | 1990-93 | 1960-64 | 1970-74 | 1980-84 | 1985-90 |
| 15-34 No. | 11 | 25 | 23 | 20 | 10 | 78 | 61 | 73 | 121 |
| Rate | 0.66 | 0.70 | 0.65 | 0.50 | 0.28 | 2.14 | 1.72 | 1.83 | 2.47 |
| 35-49 No. | 100 | 249 | 240 | 163 | 132 | 332 | 363 | 354 | 413 |
| Rate | 9.18 | 9.82 | 10.14 | 7.32 | 7 | 13.06 | 15.34 | 15.80 | 14.94 |
| 50-69 No. | 270 | 872 | 949 | 922 | 712 | 905 | 1,083 | 1,252 | 1,541 |
| Rate | 24.18 | 28.37 | 30.57 | 31.23 | 31 | 29.35 | 34.96 | 42.79 | 44.70 |
| 70-84 No. | 94 | 351 | 491 | 621 | 648 | 294 | 446 | 731 | 1,103 |
| Rate | 26.20 | 35.37 | 40.96 | 43.82 | 57 | 29.84 | 37.27 | 51.87 | 64.91 |
| Total, No. | 475 | 1,497 | 1,703 | 1,726 | 1,502 | 1,609 | 1,953 | 2,410 | 3,178 |
| 15-84 Rate | 12.17 | 14.45 | 15.70 | 15.43 | 16 | 15.53 | 18.32 | 22.19 | 24.01 |

**Table A14.** Mortality and incidence of cancer of the prostate, for selected calendar periods.

| Age | Mortality | | | | | Incidence | | | |
|---|---|---|---|---|---|---|---|---|---|
| | 1953-54 | 1960-64 | 1970-74 | 1980-84 | 1990-93 | 1960-64 | 1970-74 | 1980-84 | 1985-90 |
| 15-34 No. | 0 | 1 | 0 | 0 | 0 | 1 | 3 | 0 | 3 |
| Rate | 0.00 | 0.03 | 0.00 | 0.00 | 0.00 | 0.03 | 0.09 | 0.00 | 0.06 |
| 35-49 No. | 3 | 5 | 3 | 4 | 11 | 13 | 13 | 17 | 32 |
| Rate | 0.30 | 0.22 | 0.13 | 0.19 | 0.60 | 0.58 | 0.58 | 0.82 | 1.24 |
| 50-69 No. | 184 | 482 | 546 | 585 | 551 | 840 | 1,078 | 1,530 | 2,109 |
| Rate | 23.53 | 22.44 | 21.43 | 23.94 | 27.43 | 38.54 | 42.25 | 62.97 | 71.40 |
| 70-84 No. | 462 | 1,294 | 1,169 | 1,564 | 1,740 | 1,461 | 1,783 | 3,108 | 4,513 |
| Rate | 186.78 | 213.51 | 185.58 | 196.81 | 247.20 | 240.56 | 278.63 | 387.15 | 443.32 |
| Total, No. | 649 | 1,782 | 1,718 | 2,153 | 2,302 | 2,315 | 2,877 | 4,655 | 6,657 |
| 15-84 Rate | 24.77 | 26.97 | 23.99 | 25.82 | 31.73 | 34.42 | 39.15 | 55.64 | 63.59 |

**Table A15.** Mortality and incidence of cancer of the testis, for selected calendar periods.

| Age | Mortality | | | | | Incidence | | | |
|---|---|---|---|---|---|---|---|---|---|
| | 1953-54 | 1960-64 | 1970-74 | 1980-84 | 1990-93 | 1960-64 | 1970-74 | 1980-84 | 1985-90 |
| 15-34 No. | 25 | 63 | 83 | 50 | 19 | 138 | 222 | 348 | 462 |
| Rate | 1.72 | 1.83 | 2.36 | 1.24 | 0.55 | 3.98 | 6.31 | 8.60 | 9.17 |
| 35-49 No. | 12 | 33 | 28 | 39 | 11 | 89 | 128 | 217 | 307 |
| Rate | 1.22 | 1.34 | 1.23 | 1.68 | 1 | 3.67 | 5.65 | 9.43 | 10.45 |
| 50-69 No. | 8 | 17 | 19 | 7 | 10 | 51 | 49 | 53 | 82 |
| Rate | 1.00 | 0.65 | 0.73 | 0.28 | 1 | 1.94 | 1.87 | 2.09 | 2.68 |
| 70-84 No. | 7 | 14 | 12 | 16 | 6 | 15 | 13 | 15 | 17 |
| Rate | 2.80 | 2.30 | 1.87 | 2.01 | 1 | 2.49 | 1.93 | 1.87 | 1.72 |
| Total, No. | 52 | 127 | 142 | 112 | 46 | 293 | 412 | 633 | 868 |
| 15-84 Rate | 1.48 | 1.40 | 1.55 | 1.13 | 1 | 3.15 | 4.41 | 6.22 | 6.84 |

**Table A16.** Mortality and incidence of cancer of the bladder and urethra, for selected calendar periods.

| Sex | | Mortality | | | | | Incidence | | | |
|---|---|---|---|---|---|---|---|---|---|---|
| Age | | 1953-54 | 1960-64 | 1970-74 | 1980-84 | 1990-93 | 1960-64 | 1970-74 | 1980-84 | 1985-90 |
| **Males** | | | | | | | | | | |
| 15-34 | No. | 2 | 1 | 0 | 1 | 0 | 18 | 23 | 31 | 32 |
| | Rate | 0.12 | 0.03 | 0.00 | 0.02 | 0.00 | 0.50 | 0.65 | 0.77 | 0.65 |
| 35-49 | No. | 14 | 39 | 37 | 26 | 25 | 159 | 167 | 183 | 240 |
| | Rate | 1.38 | 1.69 | 1.65 | 1.23 | 1.36 | 6.72 | 7.44 | 8.49 | 8.90 |
| 50-69 | No. | 176 | 518 | 604 | 537 | 410 | 947 | 1,511 | 1,812 | 2,274 |
| | Rate | 20.76 | 22.70 | 23.57 | 21.74 | 20.49 | 39.55 | 58.68 | 73.50 | 76.40 |
| 70-84 | No. | 130 | 483 | 676 | 762 | 657 | 598 | 1,004 | 1,644 | 2,310 |
| | Rate | 51.52 | 79.43 | 104.35 | 94.15 | 93.21 | 98.25 | 152.84 | 201.18 | 228.17 |
| Total, | No. | 322 | 1,041 | 1,317 | 1,326 | 1,092 | 1,722 | 2,705 | 3,670 | 4,856 |
| 15-84 | Rate | 11.45 | 14.71 | 17.31 | 15.71 | 15.27 | 22.92 | 34.02 | 43.32 | 46.80 |
| **Females** | | | | | | | | | | |
| 15-34 | No. | 0 | 2 | 1 | 1 | 2 | 10 | 2 | 9 | 15 |
| | Rate | 0.00 | 0.05 | 0.03 | 0.02 | 0.06 | 0.26 | 0.06 | 0.23 | 0.30 |
| 35-49 | No. | 14 | 25 | 22 | 13 | 16 | 76 | 66 | 79 | 95 |
| | Rate | 1.31 | 1.00 | 0.93 | 0.60 | 0.83 | 3.00 | 2.79 | 3.51 | 3.48 |
| 50-69 | No. | 74 | 217 | 262 | 247 | 170 | 475 | 512 | 790 | 896 |
| | Rate | 6.93 | 7.34 | 8.32 | 8.28 | 7.26 | 15.90 | 16.31 | 26.81 | 25.69 |
| 70-84 | No. | 76 | 275 | 357 | 433 | 387 | 339 | 475 | 762 | 1,065 |
| | Rate | 21.53 | 27.35 | 29.44 | 29.92 | 32.27 | 33.99 | 39.61 | 53.22 | 61.13 |
| Total, | No. | 164 | 519 | 642 | 694 | 575 | 900 | 1,055 | 1,640 | 2,071 |
| 15-84 | Rate | 4.43 | 5.04 | 5.51 | 5.46 | 5.44 | 8.79 | 9.32 | 13.98 | 14.41 |

**Table A17.** Mortality and incidence of cancer of the eye, for selected calendar periods.

| Sex | | Mortality | | | | | Incidence | | | |
|---|---|---|---|---|---|---|---|---|---|---|
| Age | | 1953-54 | 1960-64 | 1970-74 | 1980-84 | 1990-93 | 1960-64 | 1970-74 | 1980-84 | 1985-90 |
| **Males** | | | | | | | | | | |
| 15-34 | No. | 0 | 1 | 1 | 0 | 1 | 3 | 5 | 6 | 12 |
| | Rate | 0.00 | 0.03 | 0.03 | 0.00 | 0.03 | 0.09 | 0.14 | 0.15 | 0.24 |
| 35-49 | No. | 2 | 5 | 4 | 4 | 3 | 18 | 12 | 19 | 28 |
| | Rate | 0.20 | 0.21 | 0.18 | 0.19 | 0 | 0.76 | 0.53 | 0.90 | 1.04 |
| 50-69 | No. | 5 | 14 | 19 | 21 | 5 | 49 | 57 | 64 | 92 |
| | Rate | 0.52 | 0.62 | 0.72 | 0.87 | 0 | 1.94 | 2.18 | 2.62 | 3.07 |
| 70-84 | No. | 5 | 10 | 9 | 7 | 9 | 11 | 17 | 26 | 35 |
| | Rate | 2.01 | 1.64 | 1.31 | 0.91 | 1 | 1.79 | 2.52 | 3.11 | 3.46 |
| Total, | No. | 12 | 30 | 33 | 32 | 18 | 81 | 91 | 115 | 167 |
| 15-84 | Rate | 0.39 | 0.40 | 0.39 | 0.39 | 0 | 0.97 | 1.07 | 1.35 | 1.58 |
| **Females** | | | | | | | | | | |
| 15-34 | No. | 1 | 1 | 1 | 0 | 0 | 9 | 4 | 5 | 9 |
| | Rate | 0.05 | 0.03 | 0.03 | 0.00 | 0.00 | 0.25 | 0.12 | 0.13 | 0.18 |
| 35-49 | No. | 1 | 1 | 7 | 3 | 3 | 13 | 15 | 14 | 24 |
| | Rate | 0.09 | 0.04 | 0.30 | 0.13 | 0 | 0.52 | 0.64 | 0.63 | 0.83 |
| 50-69 | No. | 4 | 14 | 21 | 17 | 6 | 44 | 52 | 55 | 72 |
| | Rate | 0.38 | 0.47 | 0.67 | 0.57 | 0 | 1.42 | 1.64 | 1.87 | 2.13 |
| 70-84 | No. | 1 | 10 | 14 | 28 | 16 | 15 | 29 | 44 | 64 |
| | Rate | 0.28 | 1.00 | 1.19 | 1.96 | 1 | 1.52 | 2.45 | 3.20 | 3.70 |
| Total, | No. | 7 | 26 | 43 | 48 | 25 | 81 | 100 | 118 | 169 |
| 15-84 | Rate | 0.18 | 0.26 | 0.40 | 0.39 | 0 | 0.78 | 0.92 | 1.06 | 1.25 |

**Table A18.** Mortality and incidence of cancer of the nervous system, for selected calendar periods.

| Sex | Mortality | | | | | Incidence | | | |
|---|---|---|---|---|---|---|---|---|---|
| Age | 1953-54 | 1960-64 | 1970-74 | 1980-84 | 1990-93 | 1960-64 | 1970-74 | 1980-84 | 1985-90 |
| **Males** | | | | | | | | | |
| 0-14 No. | 24 | 41 | 53 | 33 | 23 | 58 | 89 | 74 | 85 |
| Rate | 1.81 | 1.19 | 1.55 | 1.20 | 1.18 | 1.72 | 2.61 | 2.75 | 2.84 |
| 15-34 No. | 25 | 50 | 61 | 66 | 42 | 55 | 80 | 114 | 118 |
| Rate | 1.81 | 1.43 | 1.72 | 1.64 | 1.22 | 1.60 | 2.26 | 2.82 | 2.40 |
| 35-49 No. | 57 | 125 | 120 | 88 | 93 | 127 | 129 | 121 | 170 |
| Rate | 5.63 | 5.26 | 5.34 | 3.94 | 4.71 | 5.39 | 5.73 | 5.53 | 6.16 |
| 50-69 No. | 104 | 323 | 321 | 376 | 323 | 315 | 352 | 441 | 473 |
| Rate | 11.43 | 12.55 | 12.31 | 15.10 | 16.23 | 12.22 | 13.41 | 17.75 | 15.85 |
| 70-84 No. | 5 | 20 | 40 | 108 | 147 | 31 | 62 | 121 | 172 |
| Rate | 1.88 | 3.26 | 5.76 | 12.95 | 21.41 | 5.09 | 9.20 | 14.35 | 17.10 |
| Total, No. | 215 | 559 | 595 | 671 | 628 | 586 | 712 | 871 | 1,018 |
| 0-84 Rate | 4.62 | 4.64 | 4.95 | 5.70 | 6.56 | 4.90 | 5.92 | 7.39 | 7.19 |
| **Females** | | | | | | | | | |
| 0-14 No. | 13 | 36 | 32 | 34 | 24 | 55 | 57 | 67 | 88 |
| Rate | 1.04 | 1.10 | 0.99 | 1.29 | 1.29 | 1.68 | 1.75 | 2.68 | 3.13 |
| 15-34 No. | 16 | 43 | 50 | 39 | 29 | 69 | 58 | 73 | 89 |
| Rate | 0.97 | 1.18 | 1.41 | 0.98 | 0.87 | 1.96 | 1.63 | 1.86 | 1.88 |
| 35-49 No. | 39 | 90 | 87 | 72 | 68 | 122 | 106 | 132 | 131 |
| Rate | 3.58 | 3.52 | 3.67 | 3.19 | 3.47 | 4.75 | 4.47 | 5.81 | 4.58 |
| 50-69 No. | 62 | 238 | 250 | 302 | 227 | 263 | 256 | 341 | 352 |
| Rate | 5.47 | 7.72 | 8.08 | 10.39 | 10.06 | 8.46 | 8.26 | 11.69 | 10.13 |
| 70-84 No. | 11 | 24 | 45 | 134 | 129 | 34 | 51 | 143 | 154 |
| Rate | 3.14 | 2.44 | 3.86 | 9.82 | 11.75 | 3.49 | 4.39 | 10.39 | 9.12 |
| Total, No. | 141 | 431 | 464 | 581 | 477 | 543 | 528 | 756 | 814 |
| 0-84 Rate | 2.60 | 3.11 | 3.35 | 4.15 | 4.24 | 3.93 | 3.83 | 5.55 | 5.02 |

**Table A19.** Mortality and incidence of Hodgkin's disease, for selected calendar periods.

| Sex | Mortality | | | | | Incidence | | | |
|---|---|---|---|---|---|---|---|---|---|
| Age | 1953-54 | 1960-64 | 1970-74 | 1980-84 | 1990-93 | 1960-64 | 1970-74 | 1980-84 | 1985-90 |
| **Males** | | | | | | | | | |
| 15-34 No. | 28 | 84 | 47 | 37 | 18 | 98 | 123 | 158 | 176 |
| Rate | 1.94 | 2.47 | 1.33 | 0.91 | 0.53 | 2.85 | 3.47 | 3.94 | 3.57 |
| 35-49 No. | 29 | 87 | 45 | 35 | 12 | 77 | 81 | 98 | 85 |
| Rate | 2.84 | 3.72 | 2.01 | 1.56 | 1 | 3.30 | 3.60 | 4.36 | 2.90 |
| 50-69 No. | 31 | 128 | 80 | 41 | 24 | 131 | 132 | 92 | 99 |
| Rate | 3.43 | 5.06 | 3.08 | 1.61 | 1 | 5.24 | 5.08 | 3.60 | 3.28 |
| 70-84 No. | 7 | 27 | 26 | 30 | 27 | 32 | 40 | 49 | 52 |
| Rate | 2.88 | 4.41 | 3.92 | 3.78 | 4 | 5.22 | 5.93 | 6.13 | 5.15 |
| Total, No. | 95 | 326 | 198 | 143 | 81 | 338 | 376 | 397 | 412 |
| 15-84 Rate | 2.69 | 3.73 | 2.26 | 1.55 | 1 | 3.90 | 4.21 | 4.15 | 3.47 |
| **Females** | | | | | | | | | |
| 15-34 No. | 14 | 38 | 39 | 28 | 21 | 61 | 114 | 125 | 155 |
| Rate | 0.86 | 1.08 | 1.09 | 0.72 | 0.62 | 1.74 | 3.23 | 3.17 | 3.23 |
| 35-49 No. | 14 | 43 | 27 | 17 | 9 | 41 | 53 | 38 | 53 |
| Rate | 1.32 | 1.65 | 1.14 | 0.73 | 0 | 1.60 | 2.24 | 1.65 | 1.78 |
| 50-69 No. | 24 | 65 | 58 | 34 | 22 | 75 | 106 | 76 | 86 |
| Rate | 2.22 | 2.13 | 1.87 | 1.18 | 1 | 2.49 | 3.40 | 2.59 | 2.51 |
| 70-84 No. | 11 | 31 | 52 | 37 | 16 | 35 | 65 | 64 | 48 |
| Rate | 3.02 | 3.08 | 4.43 | 2.63 | 1 | 3.47 | 5.47 | 4.60 | 2.95 |
| Total, No. | 63 | 177 | 176 | 116 | 68 | 212 | 338 | 303 | 342 |
| 15-84 Rate | 1.58 | 1.72 | 1.65 | 1.04 | 1 | 2.09 | 3.25 | 2.76 | 2.64 |

**Table A20.** Mortality and incidence of non-Hodgkin's lymphoma, for selected calendar periods.

| Sex / Age | | Mortality | | | | | Incidence | | | |
|---|---|---|---|---|---|---|---|---|---|---|
| | | 1953-54 | 1960-64 | 1970-74 | 1980-84 | 1990-93 | 1960-64 | 1970-74 | 1980-84 | 1985-90 |
| **Males** | | | | | | | | | | |
| 15-34 | No. | 12 | 36 | 34 | 23 | 23 | 67 | 70 | 68 | 128 |
| | Rate | 0.86 | 1.04 | 0.96 | 0.57 | 0.70 | 1.97 | 1.97 | 1.68 | 2.61 |
| 35-49 | No. | 28 | 63 | 76 | 66 | 74 | 86 | 112 | 159 | 249 |
| | Rate | 2.80 | 2.67 | 3.38 | 3.03 | 4 | 3.63 | 4.98 | 7.25 | 8.98 |
| 50-69 | No. | 54 | 246 | 275 | 310 | 297 | 281 | 388 | 529 | 806 |
| | Rate | 6.33 | 10.11 | 10.65 | 12.60 | 15 | 11.35 | 15.05 | 21.24 | 26.82 |
| 70-84 | No. | 40 | 95 | 160 | 268 | 268 | 106 | 203 | 398 | 570 |
| | Rate | 15.48 | 15.67 | 24.11 | 33.05 | 38 | 17.43 | 30.68 | 48.89 | 56.46 |
| Total, | No. | 134 | 440 | 545 | 667 | 662 | 540 | 773 | 1,154 | 1,753 |
| 15-84 | Rate | 4.35 | 5.53 | 6.63 | 7.83 | 9 | 6.64 | 9.32 | 13.34 | 16.48 |
| **Females** | | | | | | | | | | |
| 15-34 | No. | 7 | 21 | 21 | 20 | 12 | 30 | 31 | 64 | 64 |
| | Rate | 0.49 | 0.59 | 0.59 | 0.51 | 0.38 | 0.84 | 0.88 | 1.62 | 1.34 |
| 35-49 | No. | 17 | 48 | 32 | 45 | 39 | 66 | 73 | 134 | 194 |
| | Rate | 1.55 | 1.87 | 1.35 | 1.98 | 2 | 2.57 | 3.08 | 5.87 | 6.87 |
| 50-69 | No. | 57 | 212 | 207 | 259 | 262 | 254 | 331 | 540 | 722 |
| | Rate | 5.17 | 6.97 | 6.59 | 8.70 | 11 | 8.38 | 10.50 | 18.16 | 20.70 |
| 70-84 | No. | 21 | 130 | 203 | 340 | 384 | 165 | 271 | 497 | 808 |
| | Rate | 5.81 | 13.03 | 16.86 | 23.67 | 33 | 16.62 | 22.52 | 34.70 | 46.94 |
| Total, | No. | 102 | 411 | 463 | 664 | 697 | 515 | 706 | 1,235 | 1,788 |
| 15-84 | Rate | 2.65 | 3.99 | 4.10 | 5.50 | 7 | 5.01 | 6.34 | 10.72 | 12.78 |

**Table A21.** Mortality and incidence of multiple myeloma, for selected calendar periods.

| Sex / Age | | Mortality | | | | | Incidence | | | |
|---|---|---|---|---|---|---|---|---|---|---|
| | | 1953-54 | 1960-64 | 1970-74 | 1980-84 | 1990-93 | 1960-64 | 1970-74 | 1980-84 | 1985-90 |
| **Males** | | | | | | | | | | |
| 15-34 | No. | 0 | 2 | 1 | 0 | 0 | 1 | 1 | 1 | 4 |
| | Rate | 0.00 | 0.05 | 0.03 | 0.00 | 0.00 | 0.03 | 0.03 | 0.02 | 0.09 |
| 35-49 | No. | 7 | 16 | 19 | 13 | 12 | 19 | 30 | 33 | 37 |
| | Rate | 0.71 | 0.70 | 0.85 | 0.61 | 0.62 | 0.83 | 1.34 | 1.55 | 1.41 |
| 50-69 | No. | 29 | 155 | 147 | 171 | 138 | 140 | 201 | 265 | 334 |
| | Rate | 3.29 | 6.38 | 5.73 | 6.96 | 6.89 | 5.64 | 7.78 | 10.75 | 11.19 |
| 70-84 | No. | 12 | 51 | 113 | 198 | 203 | 58 | 128 | 255 | 367 |
| | Rate | 4.76 | 8.39 | 17.50 | 24.25 | 29.32 | 9.50 | 19.30 | 30.92 | 36.11 |
| Total, | No. | 48 | 224 | 280 | 382 | 353 | 218 | 360 | 554 | 742 |
| 15-84 | Rate | 1.60 | 2.89 | 3.58 | 4.52 | 4.98 | 2.79 | 4.48 | 6.52 | 7.13 |
| **Females** | | | | | | | | | | |
| 15-34 | No. | 1 | 1 | 0 | 0 | 1 | 1 | 0 | 0 | 1 |
| | Rate | 0.07 | 0.03 | 0.00 | 0.00 | 0.03 | 0.03 | 0.00 | 0.00 | 0.02 |
| 35-49 | No. | 3 | 12 | 8 | 13 | 7 | 9 | 10 | 25 | 26 |
| | Rate | 0.27 | 0.48 | 0.34 | 0.59 | 0.36 | 0.36 | 0.42 | 1.14 | 0.96 |
| 50-69 | No. | 37 | 129 | 151 | 137 | 125 | 118 | 210 | 251 | 276 |
| | Rate | 3.28 | 4.29 | 4.75 | 4.64 | 5.39 | 3.93 | 6.64 | 8.40 | 7.87 |
| 70-84 | No. | 15 | 96 | 141 | 195 | 197 | 80 | 145 | 342 | 407 |
| | Rate | 4.24 | 9.63 | 11.71 | 13.49 | 16.56 | 7.98 | 12.02 | 23.91 | 23.24 |
| Total, | No. | 56 | 238 | 300 | 345 | 330 | 208 | 365 | 618 | 710 |
| 15-84 | Rate | 1.47 | 2.32 | 2.61 | 2.81 | 3.28 | 2.03 | 3.22 | 5.05 | 4.79 |

**Table A22.** Mortality and incidence of leukaemia, for selected calendar periods.

| Sex / Age | | Mortality | | | | | Incidence | | | |
|---|---|---|---|---|---|---|---|---|---|---|
| | | 1953-54 | 1960-64 | 1970-74 | 1980-84 | 1990-93 | 1960-64 | 1970-74 | 1980-84 | 1985-90 |
| **Males** | | | | | | | | | | |
| 0-14 | No. | 48 | 115 | 80 | 56 | 17 | 110 | 113 | 107 | 132 |
| | Rate | 3.73 | 3.36 | 2.33 | 2.13 | 0.86 | 3.22 | 3.31 | 4.15 | 4.45 |
| 15-34 | No. | 35 | 66 | 74 | 66 | 44 | 68 | 77 | 96 | 99 |
| | Rate | 2.62 | 1.94 | 2.09 | 1.63 | 1.52 | 1.99 | 2.18 | 2.37 | 2.05 |
| 35-49 | No. | 39 | 80 | 70 | 50 | 43 | 86 | 77 | 66 | 126 |
| | Rate | 3.85 | 3.33 | 3.11 | 2.26 | 2.16 | 3.61 | 3.42 | 2.99 | 4.56 |
| 50-69 | No. | 88 | 257 | 293 | 245 | 202 | 255 | 310 | 443 | 549 |
| | Rate | 10.22 | 10.79 | 11.34 | 9.81 | 10.13 | 10.67 | 12.02 | 17.89 | 18.43 |
| 70-84 | No. | 55 | 191 | 255 | 341 | 249 | 151 | 263 | 462 | 618 |
| | Rate | 21.67 | 31.33 | 39.62 | 42.74 | 35.46 | 24.81 | 40.38 | 57.73 | 60.58 |
| Total, | No. | 265 | 709 | 772 | 758 | 555 | 670 | 840 | 1,174 | 1,524 |
| 0-84 | Rate | 6.14 | 6.57 | 7.00 | 6.55 | 5.74 | 6.11 | 7.54 | 10.25 | 10.84 |
| **Females** | | | | | | | | | | |
| 0-14 | No. | 32 | 86 | 59 | 31 | 20 | 89 | 92 | 79 | 115 |
| | Rate | 2.59 | 2.61 | 1.81 | 1.16 | 1.06 | 2.69 | 2.84 | 3.17 | 4.05 |
| 15-34 | No. | 23 | 44 | 50 | 52 | 34 | 43 | 69 | 78 | 78 |
| | Rate | 1.53 | 1.26 | 1.42 | 1.32 | 1.06 | 1.25 | 1.95 | 1.98 | 1.64 |
| 35-49 | No. | 21 | 66 | 43 | 52 | 41 | 65 | 55 | 68 | 107 |
| | Rate | 1.91 | 2.60 | 1.81 | 2.24 | 2.04 | 2.54 | 2.33 | 2.99 | 3.77 |
| 50-69 | No. | 68 | 241 | 220 | 192 | 148 | 195 | 264 | 295 | 360 |
| | Rate | 6.30 | 8.00 | 7.03 | 6.46 | 6.43 | 6.47 | 8.38 | 9.92 | 10.30 |
| 70-84 | No. | 32 | 183 | 256 | 304 | 227 | 131 | 273 | 473 | 556 |
| | Rate | 8.94 | 18.27 | 21.27 | 20.62 | 19.04 | 13.25 | 22.65 | 32.49 | 30.91 |
| Total, | No. | 176 | 620 | 628 | 631 | 470 | 523 | 753 | 993 | 1,216 |
| 0-84 | Rate | 3.44 | 4.53 | 4.22 | 3.93 | 3.68 | 3.85 | 5.12 | 6.36 | 6.61 |

**Table A23.** Mortality and incidence of cancer of unspecified primary site, for selected calendar periods.

| Sex / Age | | Mortality | | | | | Incidence | | | |
|---|---|---|---|---|---|---|---|---|---|---|
| | | 1953-54 | 1960-64 | 1970-74 | 1980-84 | 1990-93 | 1960-64 | 1970-74 | 1980-84 | 1985-90 |
| **Males** | | | | | | | | | | |
| 15-34 | No. | 1 | 6 | 14 | 21 | 17 | 27 | 25 | 20 | 27 |
| | Rate | 0.06 | 0.17 | 0.40 | 0.51 | 0.50 | 0.77 | 0.70 | 0.49 | 0.55 |
| 35-49 | No. | 12 | 46 | 62 | 70 | 89 | 94 | 96 | 102 | 156 |
| | Rate | 1.19 | 1.93 | 2.76 | 3.28 | 4.69 | 3.99 | 4.28 | 4.74 | 5.77 |
| 50-69 | No. | 85 | 318 | 493 | 818 | 876 | 589 | 706 | 1069 | 1366 |
| | Rate | 10.03 | 13.66 | 19.15 | 33.12 | 43.81 | 24.69 | 27.37 | 43.47 | 45.94 |
| 70-84 | No. | 59 | 172 | 375 | 922 | 1,129 | 324 | 446 | 1021 | 1599 |
| | Rate | 23.77 | 28.27 | 58.24 | 113.89 | 161.70 | 53.29 | 68.18 | 125.80 | 157.65 |
| Total, | No. | 157 | 542 | 944 | 1,831 | 2,111 | 1034 | 1273 | 2212 | 3148 |
| 15-84 | Rate | 5.55 | 7.28 | 12.04 | 21.65 | 29.70 | 13.66 | 15.92 | 26.21 | 30.23 |
| **Females** | | | | | | | | | | |
| 15-34 | No. | 3 | 11 | 10 | 14 | 11 | 19 | 16 | 10 | 16 |
| | Rate | 0.17 | 0.30 | 0.28 | 0.35 | 0.33 | 0.51 | 0.45 | 0.25 | 0.32 |
| 35-49 | No. | 17 | 41 | 70 | 73 | 75 | 94 | 103 | 100 | 151 |
| | Rate | 1.62 | 1.62 | 2.95 | 3.30 | 3.94 | 3.69 | 4.35 | 4.47 | 5.48 |
| 50-69 | No. | 65 | 290 | 411 | 668 | 672 | 562 | 663 | 934 | 1256 |
| | Rate | 5.97 | 9.75 | 13.11 | 22.47 | 29.05 | 18.78 | 21.22 | 31.69 | 36.03 |
| 70-84 | No. | 71 | 268 | 528 | 1,057 | 1,209 | 390 | 641 | 1325 | 1862 |
| | Rate | 19.81 | 26.98 | 43.58 | 72.42 | 101.39 | 39.30 | 53.37 | 91.63 | 105.27 |
| Total, | No. | 156 | 610 | 1,019 | 1,812 | 1,967 | 1065 | 1423 | 2369 | 3285 |
| 15-84 | Rate | 4.11 | 5.96 | 8.86 | 14.49 | 19.34 | 10.41 | 12.61 | 19.31 | 22.17 |